# Impact of insurance type on outcomes in cardiac arrest patients from 2004 to 2015: A nation-wide population-based study

**DOI:** 10.1371/journal.pone.0254622

**Published:** 2021-07-14

**Authors:** Si Jin Lee, Kap Su Han, Eui Jung Lee, Sung Woo Lee, Myung Ki, Hyeong Sik Ahn, Su Jin Kim

**Affiliations:** 1 Department of Emergency Medicine, College of Medicine, Korea University Hospital, Seoul, South Korea; 2 Department of Preventive Medicine, College of Medicine, Korea University Hospital, Seoul, South Korea; 3 Department of Preventive Medicine, Institute for Evidence-based Medicine, The Korean Branch of Australasian Cochrane Center, College of Medicine, Korea University, Seoul, South Korea; Fondazione IRCCS Policlinico San Matteo, ITALY

## Abstract

**Objectives:**

There do not appear to be many studies which have examined the socio-economic burden and medical factors influencing the mortality and hospital costs incurred by patients with cardiac arrest in South Korea. We analyzed the differences in characteristics, medical factors, mortality, and costs between patients with national health insurance and those on a medical aid program.

**Methods:**

We selected patients (≥20 years old) who experienced their first episode of cardiac arrest from 2004 to 2015 using data from the National Health Insurance Service database. We analyzed demographic characteristics, insurance type, urbanization of residential area, comorbidities, treatments, hospital costs, and mortality within 30 days and one year for each group. A multiple regression analysis was used to identify an association between insurance type and outcomes.

**Results:**

Among the 487,442 patients with cardiac arrest, the medical aid group (13.3% of the total) had a higher proportion of females, rural residents, and patients treated in low-level hospitals. The patients in the medical aid group also reported a higher rate of non-shockable conditions; a high Charlson Comorbidity Index; and pre-existing comorbidities, such as hypertension, diabetes mellitus, and renal failure with a lower rate of providing a coronary angiography. The national health insurance group reported a lower one-year mortality rate (91.2%), compared to the medical aid group (94%), and a negative association with one-year mortality (Adjusted OR 0.74, 95% CI 0.71–0.76). While there was no significant difference in short-term costs between the two groups, the medical aid group reported lower long-term costs, despite a higher rate of readmission.

**Conclusions:**

Medical aid coverage was an associated factor for one-year mortality, and may be the result of an insufficient delivery of long-term services as reflected by the lower long-term costs and higher readmission rates. There were differences of characteristics, comorbidities, medical and hospital factors and treatments in two groups. These differences in medical and hospital factors may display discrepancies by type of insurance in the delivery of services, especially in chronic healthcare services.

## Introduction

Global incidence rates of cardiac arrest and corresponding outcomes show variations across continents due to differences in healthcare systems, race, and comorbidities [1]. The estimated number of annual out-of-hospital cardiac arrests (OHCA) occurrences in South Korea is 44 per 100,000 population [2], compared to 95 per 100,000 population in the United States, along with 200,000 patients of in-hospital cardiac arrests (IHCA) annually [3,4]. In Europe, the numbers of annual cardiac arrests occurrences ranged from 19 to 108 per 100,000 population [1,5]. The survival to hospital discharge rates in South Korea are less than 10% and 20% for OHCA and IHCA, respectively, and good neurologic survival rates vary widely, from 6–14% depending on the region [1,6–9]. High mortality rates after cardiac arrest demand time-sensitive intensive hospital care services and poor neurologic outcomes after survival demand long-term hospitalization for chronic care, thus incurring high hospital costs, despite global variations [5,10,11].

Several socioeconomic factors, such as age, sex [12], rural residence [13], and household income [14], may affect incidences and outcomes for patients with cardiovascular disease and cardiac arrest [15,16]. Factors regarding socioeconomic status, such as race, education, geographic area, employment status, and type of insurance coverage based on income, may be considered proxy measures of individual economic status [17–19]. Type of insurance coverage may influence the delivery of medical services before admission and during hospitalization, thus affecting the outcome and management of comorbidities in patients with cardiac arrest [18–22].

Health insurance service systems and the clinical characteristics of a population may affect the impact of insurance types on short- and long-term outcomes for patients with cardiac arrest. Insurance coverage may also have a financial impact on short-term hospital costs for acute care and long-term hospital costs for chronic care [1,23]. However, nation-wide studies to examine the influence of health insurance types on short- and long-term outcomes and costs rarely include patients with cardiac arrest.

The objective of this study was to analyze the impact of insurance type as the determinant on short- and long-term mortality outcomes in a universal healthcare setting, based on nation-wide population data. We also attempted to analyze differences in short- and long-term hospital costs between patients with cardiac arrest covered by national health insurance and those on a medical aid program.

## Methods

### Data source

We used cohort data from the Korean National Health Insurance Services (NHIS) claim database released by the NHIS from 2002 to 2016. The NHIS program, as a universal healthcare system, is a unique single insurer, administered by the government of Korea [24]. The NHIS, which requires all Koreans to be mandatorily registered, covers most of the citizens’ medical care and includes all medical facilities. The database contains de-identified information on all insurance claims such as age, sex, residence, an identifier for the clinic or hospital, type of insurance; diagnostic codes by the International Classification of Diseases (ICD-10, 10^th^ edition); information on reimbursements for each medical service including medications and procedures; and patient deaths [25]. The Emergency Medical Services (EMS) system is exclusively operated by the National Fire Agency. The EMS in Korea, covers most of the country, and the EMS provider cannot declare a state of death or stop cardiopulmonary resuscitation (CPR) until the patient regains a pulse. The protocol for on-scene termination of resuscitation is applied in very limited conditions for patients with obvious signs of death. EMS providers transport all OHCA patients to the emergency department (ED) under the EMS CPR protocol, even if there has been a return of spontaneous circulation (ROSC) [26,27]. This study was approved by the Institutional Review Board of Korea University Medical Center (#2017AN0083) and informed consent was waived because of the anonymous nature of the data.

### Study population

**Fig 1:**
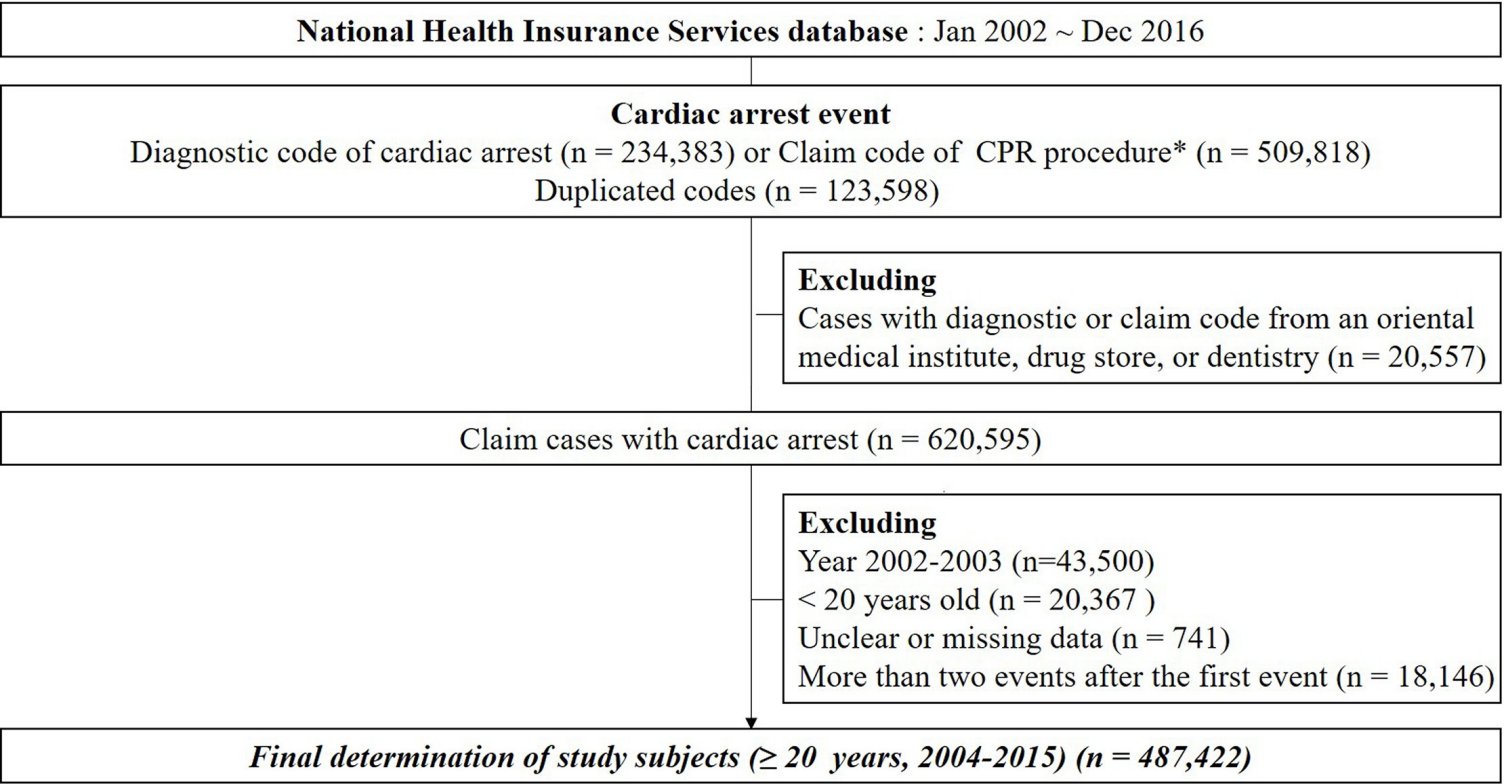
Flowchart of the selection of study population.

We identified patients with a first diagnosis of cardiopulmonary arrest during their index hospitalization based on the main-diagnosis, the 1^st^-4^th^ sub-diagnosis claims codes (I46.0–9), or the first claim codes for CPR procedures (M5871, M5873-7), and the relevant ICD-10 codes [3,4,28] using the NHIS claims data from 2004 to 2015, with a one year follow-up through 2016. Index hospitalization was defined as the first instance of hospitalization of a patient with a claim for cardiac arrest. All adult patients with cardiac arrest, aged 20 years and older, who were admitted to a hospital between 2004 and 2015 were included. The study population included all patients with IHCA and OHCA who were transported and admitted to hospital. We excluded patients below 20 years of age; those with codes from an oriental medical institute, drug store, or dentistry; and those with missing data (Fig 1).

### Definition of variables

We used raw data from the NHIS database to identify patients’ age, sex, residential areas, types of health insurance at index hospitalization; comorbidities, which were differentiated by diagnostic codes at the medical institute before index hospitalization; the Charlson Comorbidity Index (CCI) using the diagnostic codes [29]; and hospital information. The hospital levels are classified by the Ministry of Health and Welfare based on the level of medical service, function of medical care and training, human resources, and facilities etc. Tertiary-level hospitals constitute more than 20 professional departments with a resident training function, while secondary-level hospitals constitute a minimum of 100 beds with seven to nine professional departments. Urbanization level was classified based on the geographical region of the administrative divisions while considering the population.

Pre-existing comorbidities were classified [30–33], and determined when a diagnostic code was recorded at least twice within one year during visits to clinics, or when a patient had one or more hospitalizations within two years before index hospitalization. Further, we extracted details regarding specific treatments, including defibrillation, extracorporeal membrane oxygenation (ECMO), percutaneous coronary intervention (PCI), coronary angiography (CAG), coronary artery bypass graft (CABG), implantable cardioverter defibrillator (ICD) or pacemaker, continuous renal replacement therapy (CRRT), hemodialysis, electroencephalography (EEG), therapeutic hypothermia, and medications from the records of reimbursements for each medical service. The estimated total costs, length of stay in a hospital (hospitalization days), length of stay during ICU admission (ICU days), and post-hospitalization disposition were also extracted. Post-hospitalization ICU days were defined as the number of ICU days after index hospitalization.

### Insurance type

The NHIS comprises a health insurance program, which covers approximately 96% of the Korean population, and a medical aid program, which covers between 3% and 4% of the population [34]. The co-payment rate, ranging from 5% to 60%, is determined based on the severity of the disease such as cancer, cardiovascular disease, type of service, and hospital level. The medical aid program, financed by the government, provides healthcare to individuals in the lower-income group with a co-payment rate of between 0% and 15%, and is supported by the National Basic Livelihood Security System. We used the type of insurance as a proxy variable for economic status and allocated patients to either the national health insurance (NHI) or the medical aid (MA) group.

### Study outcome

The primary outcome we considered was mortality rate within 30 days (short-term) and one year (long-term) from the date of hospitalization. Secondary outcomes included analyzing the differences between short-term hospital costs during the acute care period (within 30 days of the index date) and long-term costs (from 31 days to one year after the index date). The exchange rate was assumed to be 1,055 Korean won per US $1.

### Statistical analysis

Demographic data were described using proportions for categorical variables and means with standard deviations (SD), and medians with an interquartile range (IQR) for continuous variables. We performed the Chi-square test and Fisher’s exact test for categorical variables, and the Student’s t-test, Wilcoxon rank sum test, and analysis of variance for continuous variables. In addition, we used absolute standardized differences (ASDs) to compare various characteristics between the insurance types. We analyzed ASDs as they are expected to be more informative than *p*-values while comparing large datasets; ASDs less than 0.1 represent a small standardized difference [35].

Odds ratios (OR) with 95% confidence intervals (CI) were calculated using a multiple logistic regression analysis with generalized estimating equation methods to examine the association between factors and mortality for analyzing the impact of insurance type on mortality and any change in the impact of insurance type, by adding characteristics such as age, sex, comorbidities, hospital level, urbanization, admission route, and basic treatments. All statistical analyses were performed using SAS ver. 9.4 (SAS Institute, Cary, NC).

## Results

We identified 487,442 patients with cardiac arrest, 60.2% were male, and the median age was 70 years. The NHI and MA groups accounted for 86.6% and 13.3% of the patients, respectively. Furthermore, 15.6% of the patients were rural residents; and 33.7% and 19.5% used lower-capacity (<300 beds) and primary level hospitals, respectively (Table 1).

**Table 1:** Characteristics, and hospital factors in national health insurance group and medical aid group.

	Total N (%)	NHI N (%)	MA N (%)	*p*-value	ASDs*
**Age** (years)					
20–29	8510 (1.75)	7787 (1.84)	723 (1.1)	< .0001^a^	**0.1968**
30–39	17897 (3.7)	15874 (3.8)	2023 (3.1)		
40–49	45562 (9.4)	37717 (8.9)	7845 (12.1)		
50–59	73905 (15.2)	62538 (14.8)	11367 (17.5)		
60–69	96651 (19.8)	85601 (20.3)	11050 (17.0)		
70–79	137370 (28.2)	120912 (28.6)	16458 (25.3)		
80-	107547 (22.1)	92038 (21.8)	15509 (23.9)		
Mean	66.9 ± 15.3	66.9 ± 15.3	66.7 ± 15.6	0.0026^b^	0.0128
Median (IQR^†^)	70.0 (56.0–78.0)	70.0 (57.0–78.0)	69.0 (54.0–79.0)		
**Gender**					
Female	193865 (39.8)	164077 (38.8)	29788 (45.9)	< .0001^c^	**0.1422**
Male	293577 (60.2)	258390 (61.2)	35187 (54.2)		
**Urbanization level**					
Urban	410484 (84.4)	358319 (85.0)	52165 (80.4)	< .0001^c^	**0.1217**
Rural	76033 (15.6)	63307 (15.0)	12726 (19.6)		
**Level of Hospital**					
Tertiary	155106 (31.8)	140759 (33.3)	14347 (22.1)	< .0001^a^	**0.2994**
Secondary	237272 (48.7)	204921 (48.5)	32351 (49.8)		
Primary	95064 (19.5)	76787 (18.2)	18277 (28.1)		
**Capacity of hospital** (beds)					
<300	164083 (33.7)	135424 (32.1)	28659 (44.1)	< .0001^a^	**0.2826**
300–499	86446 (17.7)	74589 (17.7)	11857 (18.3)		
500–799	149424 (30.7)	132560 (31.4)	16864 (26.0)		
>800	87489 (18.0)	79894 (18.9)	7595 (11.7)		
**Admission route** (ER^‡^)	336046 (68.9)	297257 (70.4)	38789 (59.7)	< .0001^a^	**0.2250**
**Total**	487442(100.0)	422467 (86.7)	64975 (13.3)		

^a^Chi-square test

^b^Student’s t-test

^c^Fisher’s exact test.

ASDs*, Absolute standardized differences; IQR^†^, interquartile range; ED^‡^, Emergency room.

### Characteristics and hospital factors

The MA group showed a higher proportion of patients over 80 years of age, females, rural residents, and patients using low-level and low-capacity hospitals when compared to the NHI group. The rate of admission through emergency rooms (ER) was lower in the MA group than in the NHI group (Table 1).

The MA group reported a significantly higher rate of patients with ischemic stroke, chronic respiratory disease, chronic renal failure, liver cirrhosis, hypertension, and diabetes mellitus (DM). The MA group also reported a higher CCI than the NHI group (Table 2). Such as the results of Tables 1 and 2, demographic, hospital factors, comorbidities were shown to be consistent tendency in the cardiac arrest patients who admitted through ER (S1 Table).

**Table 2:** Comorbidities in national health insurance group and medical aid group.

	Total	NHI	MA	*p*-value	ASDs*
**CCI**^†^					
Median (IQR^‡^)	2.0 (1.0–5.0)	2.0 (1.0–5.0)	3.0 (1.0–5.0)	< .0001^a^	**0.2017**
CCI <2	178778 (36.7)	162395 (38.4)	16383 (25.2)	< .0001^b^	0.2868
**Comorbidities,** n (%)					
Cancer	100616 (20.6)	88593 (21.0)	12023 (18.5)	< .0001^b^	0.0620
Ischemic stroke	92087 (18.9)	76470 (18.1)	15617 (24.0)	< .0001^b^	**0.1459**
Hemorrhagic stroke	17837 (3.7)	14680 (3.5)	3157 (4.9)	< .0001^b^	0.0693
Myocardial infarction	19218 (3.94)	16528 (3.91)	2690 (4.1)	0.0058^b^	0.0116
Angina	68278 (14.0)	59131 (14.0)	9147 (14.1)	0.5806^b^	0.0023
Heart failure	52117 (10.7)	44014 (10.4)	8103 (12.5)	< .0001^b^	0.0645
Arrhythmia	45377 (9.31)	39233 (9.29)	6144 (9.5)	0.1683^b^	0.0058
Hypertension (HTN)	258264 (53.0)	220955 (52.3)	37309 (57.4)	< .0001^b^	**0.1030**
Diabetes Mellitus (DM)	155281 (31.9)	131049 (31.0)	24232 (37.3)	< .0001^b^	**0.1326**
Lipidemia	113895 (23.4)	97891 (23.2)	16004 (24.6)	< .0001^b^	0.0342
Chronic pulmonary disease	146034 (30.0)	122432 (29.0)	23602 (36.3)	< .0001^b^	**0.1571**
Chronic renal failure	37686 (7.7)	30588 (7.2)	7098 (10.9)	< .0001^b^	**0.1285**
Hemodialysis	20948 (4.3)	16879 (4.0)	4069 (6.3)	< .0001^b^	**0.1029**
Liver cirrhosis	90018 (18.5)	73824 (17.5)	16194 (24.9)	< .0001^b^	**0.1830**

^a^Wilcoxon rank sum test

^b^Fisher’s exact test.

ASDs*, Absolute standardized differences; CCI^†^, Charlson Comorbidity Index; IQR^‡^, interquartile range.

### Medications and procedures

Defibrillation at the hospital was conducted in 26.5% of the total number of cases, and was more frequent in the NHI group (27.1%) than in the MA group (22.7%). Amiodarone and coronary angiogram (CAG) were more frequently provided to the NHI group than the MA group. Specific treatments and procedures, including percutaneous coronary intervention (PCI), coronary artery bypass graft surgery (CABG), veno-arterial extracorporeal membrane oxygenation (ECMO), continuous renal replacement therapy (CRRT), and therapeutic hypothermia did not show significant ASDs in both groups. The long-term mortality rate was higher in the MA group at 94% as opposed to 91.2% in the NHI group. The short-term mortality rate was 84.9% in the MA group but the ASDs were not statistically significant (Table 3).

**Table 3:** Medications and procedures in national health insurance group and medical aid group.

	Total N (%)	NHI N (%)	MA N (%)	*p*-value	ASDs
Defibrillation	129060 (26.5)	114302 (27.1)	14758 (22.7)	< .0001^a^	**0.1006**
Epinephrine	396146 (81.3)	345054 (81.7)	51092 (78.6)	< .0001^a^	0.0763
Amiodarone	56220 (11.5)	50699 (12.0)	5521 (8.5)	< .0001^a^	**0.1157**
Atropine	292674 (60.0)	254330 (60.2)	38344 (59.0)	< .0001^a^	0.0242
CAG*	26545 (5.5)	24397 (5.8)	2148 (3.3)	< .0001^a^	**0.1188**
PCI^†^	16004 (3.3)	14742 (3.5)	1262 (1.9)	< .0001^a^	0.0953
CABG^‡^	1344 (0.28)	1248 (0.30)	96 (0.15)	< .0001^a^	0.0314
ECMO^**§**^	4376 (0.9)	4155 (1.0)	221 (0.3)	< .0001^a^	0.0794
ICD^**||**^	1107 (0.23)	1060 (0.25)	47 (0.1)	< .0001^a^	0.0445
CRRT^**^	28123 (5.8)	24991 (5.9)	3132 (4.8)	< .0001^a^	0.0486
Brain CT	104982 (21.5)	91924 (21.8)	13058 (20.1)	< .0001^a^	0.0409
Brain MRI	20140 (4.1)	17845 (4.2)	2295 (3.5)	< .0001^a^	0.0358
EEG^¶^	22280 (4.6)	19866 (4.7)	2414 (3.7)	< .0001^a^	0.0492
Therapeutic hypothermia	5220 (1.1)	4896 (1.2)	324 (0.5)	< .0001^a^	0.0729
30-ds death	411375 (84.4)	356185 (84.3)	55190 (84.9)	< .0001^a^	0.0175
6-mo death	442253 (90.7)	381882 (90.4)	60371 (92.9)	< .0001^a^	0.0912
1-yr death	446414 (91.6)	385337 (91.2)	61077 (94.0)	< .0001^a^	**0.1068**

^a^Fisher`s exact test.

CAG*, Coronary angiography; PCI^†^, Percutaneous coronary intervention; CABG^‡^, Coronary artery bypass graft; ECMO^**§**^, Veno-arterial extracorporeal membrane oxygenation; ICD^**||**^, Implanted cardioverter-defibrillator, ETCO_2_^**#**^, End-tidal carbon dioxide; CRRT^**^, Continuous renal replacement therapy; EEG^¶^, Electroencephalography.

### Adjusted Odds ratio of insurance type for mortality depending on the adjusting factors (Table 4)

After adjusting the age group, gender, CCI, admission route through the ER, hospital level, level of urbanization, provision of defibrillation, and epinephrine medication, the type of health insurance coverage was found to be negatively associated with one-year mortality (adjusted OR, 0.74; 95% CI 0.71–0.76). When adjusting for age group, gender, CCI, and admission route factors, health insurance coverage was found to be negatively associated with 30-days mortality (adjusted OR, 0.91; 95% CI 0.89–0.93). However, insurance type did not report any association with 30-days mortality (adjusted OR, 0.99; 95% CI 0.97–1.02) after adjusting for hospital level, level of urbanization, and resuscitation-related treatments (*p* = 0.519) in Table 4A. While NHI coverage did not show any association with 30-days mortality (adjusted OR, 0.99; 95% CI 0.96–1.02; *p* = 0.390) in cardiac arrest patients who were admitted through ER, NHI coverage was negatively associated with one-year mortality (adjusted OR, 0.737; 95% CI 0.71–0.77; *p*<0.0001) in Table 4B.

**Table 4:** Factors independently associated with short-term and long-term mortality outcome.

A. Adjusted ORs* for mortality outcome in the patients with cardiac arrest				
	30-days mortality		1-year mortality	
Variables	Adjusted ORs (95% CI^**†**^)	*p*-value	Adjusted ORs (95% CI^**†**^)	*p*-value
Age (per 10 years)	1.190 (1.184, 1.196)	< .0001	1.296 (1.288,1.303)	< .0001
Male	1.050 (1.033, 1.067)	< .0001	1.110 (1.087,1.134)	< .0001
CCI^**§**^	1.004 (1.002, 1.007)	0.0011	1.071 (1.067,1.075)	<0001
Admission route (ER^**‡**^)	1.397 (1.373, 1.421)	< .0001	1.013 (0.989,1.037)	0.2911
**National Health insurance**	0.913 (0.892, 0.934)	< .0001	0.687 (0.663,0.771)	< .0001
Age (per 10 years)	1.154 (1.148, 1.159)	< .0001	1.270 (1.263, 1.278)	< .0001
Male	1.060 (1.042, 1.077)	< .0001	1.112 (1.089, 1.136)	< .0001
CCI^**§**^	1.009 (1.007, 1.012)	< .0001	1.075 (1.071, 1.079)	< .0001
Admission route (ER^**‡**^)	1.759 (1.727, 1.791)	< .0001	1.171 (1.142, 1.201)	< .0001
*Level of hospital (Primary)* Ref			Ref	
Tertiary	0.384 (0.373, 0.394)	< .0001	0.496 (0.478, 0.514)	< .0001
Secondary	0.467 (0.455, 0.480)	< .0001	0.624 (0.602, 0.647)	< .0001
*Urbanization level (Rural)*	Ref		Ref	
City	1.030 (1.007, 1.054)	0.0094	1.059 (1.028, 1.091)	0.0002
**National Health insurance**	0.982 (0.959, 1.005)	**0.1307**	0.728 (0.703, 0.753)	< .0001
Age (per 10 years)	1.157 (1.151, 1.163)	< .0001	1.279 (1.271, 1.286)	< .0001
Male	1.056 (1.038, 1.073)	< .0001	1.112 (1.089, 1.137)	< .0001
CCI^**§**^	1.014 (1.011, 1.017)	< .0001	1.083 (1.079, 1.087)	< .0001
Admission route (ER^**‡**^)	1.726 (1.694, 1.759)	< .0001	1.126 (1.097, 1.156)	< .0001
*Level of hospital (Primary)*	Ref		Ref	
Secondary	0.413 (0.401, 0.424)	< .0001	0.520 (0.501, 0.540)	< .0001
Tertiary	0.326 (0.317, 0.336)	< .0001	0.389 (0.374, 0.405)	< .0001
*Urbanization level (Rural)*	Ref		Ref	
Urban	1.021 (0.998, 1.044)	0.0767	1.042 (1.011, 1.073)	0.0081
Defibrillation	0.821 (0.806, 0.836)	< .0001	0.678 (0.663, 0.694)	< .0001
Epinephrine	1.954 (1.914, 1.994)	< .0001	2.934 (2.859, 3.011)	< .0001
**National Health insurance**	0.992 (0.969, 1.016)	**0.5192**	0.737 (0.712, 0.764)	< .0001
B. Adjusted ORs* **for mortality outcome in the patients with cardiac arrest who were admitted through ER**				
	**30-days mortality**			**1-year mortality**
	Adjusted ORs* (95% CI^**†**^)	*p*-value	Adjusted ORs* (95% CI^**†**^)	*p*-value
Age (years)	1.019 (1.018, 1.019)	< .0001	1.029 (1.029, 1.030)	< .0001
Male	1.037 (1.016, 1.059)	0.0006	1.075 (1.047, 1.103)	< .0001
*Level of Hospital*				
Primary	Ref		Ref	
Secondary	0.608 (0.583, 0.635)	< .0001	0.629 (0.596, 0.663)	< .0001
Tertiary	0.476 (0.455, 0.497)	< .0001	0.475 (0.449, 0.502)	< .0001
*Urbanization level (Rural)*	Ref		Ref	
Urban	1.034 (1.002, 1.066)	0.0365	1.074 (1.033, 1.117)	0.0004
CCI^**§**^≥ 2	1.016 (0.990, 1.044)	0.2331	1.256 (1.212, 1.302)	< .0001
Defibrillation	0.816 (0.798, 0.834)	< .0001	0.675 (0.657, 0.694)	< .0001
Epinephrine	2.758 (2.680, 2.838)	< .0001	3.374 (3.259, 3.492)	< .0001
**National Health insurance**	0.986 (0.956, 1.018)	**0.3906**	0.737 (0.706, 0.770)	< .0001

*ORs*, Odds ratios; CI^†^, Confidence intervals; ER^**‡**^, Emergency room; CCI^**§**^, Charlson Comorbidity Index.

### Length of stay, hospital costs per person, and post-hospitalization disposition

The median number of hospitalization days was 2 (1–11) days in the NHI group and 4 (1–14) days in the MA group. The median intensive care unit (ICU) days during index hospitalization was 4 (1–10) days and 4 (2–12) days in the NHI and MA groups, respectively.

There was no significant difference between the short-term hospital costs per person for patients in the NHI group ($1,302) and patients in the MA group ($1,384). However, the MA group reported lower long-term hospital costs at $7,279 per person compared to $8,737 per person for the NHI group (Table 5).

**Table 5:** Length of stay, hospital costs, and post-hospitalization disposition in national health insurance group and medical aid group.

	Total	NHI	MA	*p*-value	ASDs
**Hospitalization days**					
Median (IQR*)	3.0 (1.0–11.0)	2.0 (1.0–11.0)	4.0 (1.0–14.0)	< .0001^a^	**0.1190**
Mean ± SD^**†**^	8.8 ± 15.1	8.6 ± 15.0	10.4 ± 15.8		
N	487442	422467	64975		
**ICU**^**‡**^ **days**					
Median (IQR*)	4.0 (1.0–11.0)	4.0 (1.0–10.0)	4.0 (2.0–12.0)	< .0001^a^	0.0603
Mean ± SD^**†**^	8.4 ± 13.0	8.3 ± 13.0	9.1 ± 12.9		
N ^b^	200076	173748	26328		
**Post-hospitalization ICU days**					
Median (IQR*)	9.0 (3.0–24.0)	8.0 (3.0–23.0)	10.0 (3.0–28.0)	< .0001^a^	0.0736
Mean ± SD^**†**^	23.1 ± 44.8	22.7 ± 44.6	26.0 ± 45.7		
N	27890	24455	3435		
**Hospital cost/person,** Median (IQR*)					
Short-term Hospital cost	$1315 (431–4357)	$1302 (426–4442)	$1384 (473–3915)	0.4451^a^	0.0974
Mean ± SD^**†**^	$3617 ± 5841	$3686 ± 6003	$3165 ± 4620		
N	487441	422467	64975		
Long-term Hospital cost	$8516 (2973–23404)	$8737 (3087–24026)	$7279 (2404–20013)	< .0001^a^	**0.1657**
Mean ± SD^**†**^	$17228± 22854	17755 ± 23595	14285 ± 17885		
N ^c^	47580	40358	7222		
**Post-hospitalization disposition**, n (%)					
Continuing Admission	39168 (8.0)	34068 (8.1)	5100 (7.9)	< .0001^d^	**0.1061**
Readmission	3552 (0.7)	2543 (0.6)	1009 (1.6)		
Outpatient clinic	48329 (9.9)	42837 (10.1)	5492 (8.5)		

^a^Wilcoxon rank sum test

^b^The number of patients who were admitted to the ICU during index hospitalization

^c^The number of patients who had been hospitalized from 31 days to one year

^d^Chi-square test.

IQR*, interquartile range; SD^**†**^, standard deviation; ICU^**‡**^, Intensive care unit.

## Discussion

We identified 487,442 patients from 2004 to 2015 (about 30,000–50,000 cardiac arrest patients hospitalized annually, including both OHCA and IHCA among a total population of nearly 49 million in 2010, according to the Korea National Census database [36]. Several studies reported the rate of survival at discharge as 14%–22% for IHCA [9] and 2%-9% for OHCA [1,3], while our study reported a 30-day mortality rate of 84.4% and one-year mortality rate of 91.6% for both OHCA and IHCA. The NHIS is a single, universal health insurer under government supervision, which covers almost the entire population of South Korea, while nearly 1.5 million Koreans (3% of the population) are covered by the medical aid program. The NHIS provides most of the necessary care at a low cost. The medical aid program is a surrogate for information regarding individuals at the lowest economic level in the national healthcare program. The proportion of patients with cardiac arrests in the MA group was 13.3%, which is higher than the proportion of the national population using medical aid (3%). Cardiac arrest was more common in the MA group and Kim et al. also reported that patients with medical aid as their health insurance coverage were less likely to receive post-resuscitation care and had poorer outcomes when compared with the NHI group [26].

Consistent with past studies, the MA group reported a higher number of patients aged 80 and older, females, and rural residents compared to the NHI group [18,37]. These differences may be attributed to the fact that the MA group comprises patients with low household income and that are supported by the National Basic Livelihood Security System.

Moreover, the MA group depicted a higher comorbidity burden (CCI ≥ 2), and a higher rate of comorbidities, such as ischemic stroke, hypertension, diabetes mellitus, chronic pulmonary disease, chronic renal failure, and liver cirrhosis, but not cardiogenic diseases. Several studies reported that the higher the rate of stroke [26,37,38], hypertension [18,38], DM [18,26,38], chronic renal failure [18,38], and chronic pulmonary disease [18,38], the higher the comorbidity burden [38] in patients with cardiac arrest and cardiovascular risk in the MA group, which is similar to our results. Although the healthcare insurance system depicts regional differences, a lack of health insurance has been associated with increasing rates of stroke and death, less control of cardiovascular risk conditions, and mortality after myocardial infarction [37,38]. The incidence of sudden cardiac arrest is twice as high in the lowest quartile by median household income of OHCA patients; furthermore, there is a higher incidence of cardiac arrest and an increasing number of cardiovascular disease risk factors in the low socioeconomic group affecting health-promoting behavior, especially among patients below 65 years of age [15,39].

Patients in the MA group reported a higher likelihood of being admitted to primary level hospitals, lower number of cases admitted through the ER, and a lower number of high-cost special services like CAG. Medical aid coverage showed differences in accessibility to high-level, large-capacity hospitals with emergency care, and delivery of high-cost special services and treatments.

A lack of adequate insurance coverage or economic status may influence access to healthcare or delivery of services after admission [14,21,39]. Hospitals with higher case volumes and cardiac-specific facilities reported higher survival rates when affiliated to academic medical centers [40]. Although the selected population and defined outcome varied, non-private insurance, such as Medi-care or medical aid coverage, also reported a positive association with poor neurologic outcomes at discharge and a lower rate of post-arrest care treatment like PCI at 24/7 PCI centers [18,19,22,26].

The rate of defibrillation and amiodarone medications was lower in the MA group. The MA group may also have had a higher rate of non-shockable rhythm due to more severe non-cardiac comorbidities. Eid et al. and Coppler et al. reported that a high comorbidity burden and non-shockable rhythm show a positive association with short- and long-term mortality [22,23]. Such different pre-existing conditions can induce the results of a lower rate of defibrillation, given amiodarone, and provided CAG due to chronic non-cardiac diseases.

There was no association between the short-term mortality rate and the MA program after adjusting for other factors, and the short-term cost was similar in both groups, despite reports of longer hospitalization in the MA group. The impact of the MA program on short-term mortality was diminished after adjusting for hospital and urbanization levels.

MA coverage was positively associated with a long-term mortality rate. After adjusting for demographics, hospital factors, and comorbidities, the analyses reported differences in long-term outcomes based on the type of insurance, despite social support by the NHIS system, which covers most medical services. The MA group’s coverage status was found to be an associated factor for long-term mortality after adjusting for age, gender, CCI, cases admitted through ER, hospital level, urbanization level, and provision of defibrillation and epinephrine medication. Although Uray et al. reported no association between good neurologic outcomes and insurance status in cardiac arrest patients under the age of 65 years [21], several studies reported an association between a lack of medical insurance and mortality and poor neurologic outcome at discharge in cardiac arrest patients [18,19,26,38]. The association between lack of insurance and poor outcomes may be multifaceted and is likely a result of poor access to regular health care and special care, an unhealthy lifestyle, and differences in the delivery of services after admission.

Although we could not clearly divide the population into IHCA and OHCA groups, we could analyze hospitalization admission routes as a surrogate for OHCA and IHCA, according to the admission route through ER. The study found that 68.9% of the patients were the cardiac arrest patients with admission through ER, and 31.1% were patients without admission through non-emergency room. Similar results in terms of differences in demographics, hospital factors, comorbidities, basic treatments, and mortality between NHI and MA groups were shown in the patients admitted through ER (S1 Table).

Several studies reported that IHCA showed lower mortality compared to OHCA due to a more rapid response and start of CPR by health care providers, shorter time to defibrillate in a shockable rhythm, and shorter interval from collapse to starting ECMO or PCI [41–43]. In this study, the adjusted OR for “admission route through ER” was 1.726 (95% CI 1.694–.759) for 30-day mortality and 1.126 (95% CI 1.097–1.156) for one-year mortality after adjusting for demographic factors, hospital factors, CCI, and basic treatments. The patients admitted through ER showed a lower rate of comorbidities and a higher 30-day mortality rate than the patients not admitted through ER (S2 Table).

The use of epinephrine is also positively associated with 30-day mortality (aOR 1.954, 95% CI 1.914–1.994) and one-year mortality (aOR 2.934, 95% CI 2.859–3.011). Dumas et al. reported that the use of epinephrine was associated with a lower chance of survival among cardiac arrest patients because epinephrine could be considered a surrogate marker of disease severity, and its vasoconstrictive action may promote secondary detrimental effects during the post-cardiac arrest phase, combining myocardial dysfunction, ischemia-reperfusion, and post-anoxic injury [44]. Similar to our results, a higher survival rate in high-level hospitals or academic medical centers was reported due to their ability to facilitate intensive post-arrest care [40,45].

The MA group showed longer hospitalization, both groups reported similar short-term hospital costs. The MA group reported lower long-term hospital costs compared to the NHI group, despite a higher rate of readmission. Medical aid coverage may influence short-term costs for acute care such as coronary angiography, and long-term hospital costs for chronic care. The difference in hospital costs may reflect the difference in delivery of services, especially in chronic healthcare service, due to type of insurance. This economic burden during the chronic period may impact the long-term outcome for chronic periods [11,46].

## Limitations

Since our data were based on the insurance claim database and data on private insurance coverage, resuscitation-related variables including Utstein-style pre-hospitalization information could not be included. Therefore, there may be unidentified confounders affecting outcomes. The in-hospital variables were obtained using secondary diagnostic codes and operational procedure codes, which lack the detailed clinical information recorded during index hospitalization. The lack of clinical information in relation to time flow such as procedure, medication use, and laboratory values may not exclude the influence of known confounders. Thus, the scope for establishing a causal relationship between the clinical characteristics and outcomes is limited. As the mortality rate on the day of arrest was high in patients suffering from cardiac arrest, we need to consider that non-survivors did not have a chance to receive treatment when analyzing the differences in providing post-arrest treatments between the two groups. Moreover, data regarding survival with a functional recovery among hospitalized OHCA patients were not available.

Since we included both IHCA and OHCA patients, the origin of patients could not be determined. We may divide the population into IHCA and OHCA patients by the admission route through ER, as a surrogate for OHCA and IHCA. The patients admitted through ER showed similar differences in characteristics and hospital factors between NHI and MA groups (S1 Table). As several studies reported that OHCA and IHCA showed differences in characteristics and mortality [41–43], further analysis focused on OHCA or IHAC would be required.

The data regarding hospital costs also did not include information on other types of chronic supportive care for outpatient rehabilitation, nursing homes, pre-hospitalization management (including emergency medical service system costs), or the indirect costs of returning to ordinary life. We also did not measure the cost per survivor per life year gained. Moreover, all costs for medical procedures performed in prehospitalization areas and during ambulance transportation are covered by the tax-based EMS operation budget and not charged to patients. The health insurance claim data did not include pre-hospitalization medical costs.

Despite the advantages of using nationwide, in-hospital healthcare insurance data on an individual level regarding information for all patients with cardiac arrest, insurance data is subject to coding error, and omission of costs and areas of private healthcare are not covered by the NHIS. Our results showed similar trends as other reports on long-term, continuing hospitalization in Asia [41]. However, generalizability to other countries may be limited due to different healthcare insurance systems and cultural factors.

Lastly, in this study using administrative database, it is difficult to explain the causal relationships of lack of insurance with poor outcome differed in terms of 30-day and one-year mortality. As subsequent healthcare decisions are complex, further research with large clinical data is needed.

## Conclusions

Medical aid coverage was found to be an associated factor for one-year mortality, but not for short-term mortality after adjusting for demographic, medical, and hospital factors under the national insurance coverage system. Although the MA group showed longer hospitalization, both groups reported similar short-term hospital costs. That said, the MA group reported lower long-term hospital costs, despite a higher rate of readmission. The differences in hospital factors and costs may display discrepancies by the type of insurance in the delivery of services, especially in chronic healthcare service. Since this study was based on an administrative claims database, it was difficult to establish causal relationships between factors. To overcome the limitations regarding the database’s characteristics, future research should be based on a larger registry-based data with clinical information.

## Supporting information

S1 Table Characteristics, hospital factors, treatments, and outcomes according to type of insurance coverage in cardiac arrest patients admitted through emergency room. (DOCX)

S2 Table The characteristics, comorbidities, and mortality between both groups by admission route. (DOCX)

## References

[1] Berdowski J, Berg RA, Tijssen JG, Koster RW. Global incidences of out-of-hospital cardiac arrest and survival rates: Systematic review of 67 prospective studies. Resuscitation. 2010;81(11):1479–87. Epub 2010/09/11. doi: 10.1016/j.resuscitation.2010.08.006 . 20828914

[2] Sudden cardiac arrest Survey. The Korean Centers for Disease Control and Prevention, Ministry of Health Welfare and Family Affairs of Korea. http://kosis.kr/statHtml/statHtml.do?orgId=117&tblId=DT_117088N_01&conn_path=I2.

[3] Merchant RM, Yang L, Becker LB, Berg RA, Nadkarni V, Nichol G, et al. Incidence of treated cardiac arrest in hospitalized patients in the United States. Critical care medicine. 2011;39(11):2401–6. Epub 2011/06/28. doi: 10.1097/CCM.0b013e3182257459 ; PubMed Central PMCID: PMC3196742. 21705896 PMC3196742

[4] Nichol G, Thomas E, Callaway CW, Hedges J, Powell JL, Aufderheide TP, et al. Regional variation in out-of-hospital cardiac arrest incidence and outcome. Jama. 2008;300(12):1423–31. Epub 2008/09/25. doi: 10.1001/jama.300.12.1423 ; PubMed Central PMCID: PMC3187919. 18812533 PMC3187919

[5] Graf J, Muhlhoff C, Doig GS, Reinartz S, Bode K, Dujardin R, et al. Health care costs, long-term survival, and quality of life following intensive care unit admission after cardiac arrest. Critical care (London, England). 2008;12(4):R92. Epub 2008/07/22. doi: 10.1186/cc6963 ; PubMed Central PMCID: PMC2575575. 18638367 PMC2575575

[6] Sasson C, Rogers MA, Dahl J, Kellermann AL. Predictors of survival from out-of-hospital cardiac arrest: a systematic review and meta-analysis. Circulation Cardiovascular quality and outcomes. 2010;3(1):63–81. Epub 2010/02/04. doi: 10.1161/CIRCOUTCOMES.109.889576 . 20123673

[7] Goldberger ZD, Chan PS, Berg RA, Kronick SL, Cooke CR, Lu M, et al. Duration of resuscitation efforts and survival after in-hospital cardiac arrest: an observational study. Lancet (London, England). 2012;380(9852):1473–81. Epub 2012/09/11. doi: 10.1016/S0140-6736(12)60862-9 ; PubMed Central PMCID: PMC3535188. 22958912 PMC3535188

[8] Reynolds JC, Frisch A, Rittenberger JC, Callaway CW. Duration of resuscitation efforts and functional outcome after out-of-hospital cardiac arrest: when should we change to novel therapies? Circulation. 2013;128(23):2488–94. Epub 2013/11/19. doi: 10.1161/CIRCULATIONAHA.113.002408 ; PubMed Central PMCID: PMC4004337. 24243885 PMC4004337

[9] Girotra S, Nallamothu BK, Spertus JA, Li Y, Krumholz HM, Chan PS. Trends in survival after in-hospital cardiac arrest. The New England journal of medicine. 2012;367(20):1912–20. Epub 2012/11/16. doi: 10.1056/NEJMoa1109148 ; PubMed Central PMCID: PMC3517894. 23150959 PMC3517894

[10] Naess AC, Steen PA. Long term survival and costs per life year gained after out-of-hospital cardiac arrest. Resuscitation. 2004;60(1):57–64. Epub 2004/02/28. doi: 10.1016/S0300-9572(03)00262-4 . 14987785

[11] Chan PS, Nallamothu BK, Krumholz HM, Curtis LH, Li Y, Hammill BG, et al. Readmission rates and long-term hospital costs among survivors of an in-hospital cardiac arrest. Circulation Cardiovascular quality and outcomes. 2014;7(6):889–95. Epub 2014/10/30. doi: 10.1161/CIRCOUTCOMES.114.000925 ; PubMed Central PMCID: PMC4241155. 25351479 PMC4241155

[12] Hagihara A, Onozuka D, Ono J, Nagata T, Hasegawa M. Age x Gender Interaction Effect on Resuscitation Outcomes in Patients With Out-of-Hospital Cardiac Arrest. The American journal of cardiology. 2017;120(3):387–92. Epub 2017/06/04. doi: 10.1016/j.amjcard.2017.05.003 . 28576267

[13] Masterson S, Wright P, O’Donnell C, Vellinga A, Murphy AW, Hennelly D, et al. Urban and rural differences in out-of-hospital cardiac arrest in Ireland. Resuscitation. 2015;91:42–7. Epub 2015/03/31. doi: 10.1016/j.resuscitation.2015.03.012 . 25818707

[14] Clarke SO, Schellenbaum GD, Rea TD. Socioeconomic status and survival from out-of-hospital cardiac arrest. Academic emergency medicine: official journal of the Society for Academic Emergency Medicine. 2005;12(10):941–7. Epub 2005/10/06. doi: 10.1197/j.aem.2005.05.031 . 16204138

[15] Choiniere R, Lafontaine P, Edwards AC. Distribution of cardiovascular disease risk factors by socioeconomic status among Canadian adults. CMAJ: Canadian Medical Association journal = journal de l’Association medicale canadienne. 2000;162(9 Suppl):S13–24. Epub 2000/05/17. ; PubMed Central PMCID: PMC1232440. 10813023 PMC1232440

[16] Reinier K, Stecker EC, Vickers C, Gunson K, Jui J, Chugh SS. Incidence of sudden cardiac arrest is higher in areas of low socioeconomic status: a prospective two year study in a large United States community. Resuscitation. 2006;70(2):186–92. Epub 2006/07/04. doi: 10.1016/j.resuscitation.2005.11.018 . 16814445

[17] Park CH, Ahn KO, Shin SD, Park JH, Lee SY. Association between health insurance status and transfer of patients with return of spontaneous circulation after out-of-hospital cardiac arrest. Resuscitation. 2020;149:143–9. Epub 2020/03/03. doi: 10.1016/j.resuscitation.2020.02.018 . 32114072

[18] Niedzwiecki MJ, Hsia RY, Shen YC. Not All Insurance Is Equal: Differential Treatment and Health Outcomes by Insurance Coverage Among Nonelderly Adult Patients With Heart Attack. Journal of the American Heart Association. 2018;7(11). Epub 2018/06/07. doi: 10.1161/JAHA.117.008152 ; PubMed Central PMCID: PMC6015377. 29871858 PMC6015377

[19] Casey SD, Mumma BE. Sex, race, and insurance status differences in hospital treatment and outcomes following out-of-hospital cardiac arrest. Resuscitation. 2018;126:125–9. Epub 2018/03/09. doi: 10.1016/j.resuscitation.2018.02.027 ; PubMed Central PMCID: PMC5899667. 29518439 PMC5899667

[20] Lee SY, Song KJ, Shin SD, Ro YS, Hong KJ, Kim YT, et al. A disparity in outcomes of out-of-hospital cardiac arrest by community socioeconomic status: A ten-year observational study. Resuscitation. 2018;126:130–6. Epub 2018/02/27. doi: 10.1016/j.resuscitation.2018.02.025 . 29481908

[21] Uray T, Mayr FB, Fitzgibbon J, Rittenberger JC, Callaway CW, Drabek T, et al. Socioeconomic factors associated with outcome after cardiac arrest in patients under the age of 65. Resuscitation. 2015;93:14–9. Epub 2015/05/25. doi: 10.1016/j.resuscitation.2015.04.032 ; PubMed Central PMCID: PMC4856150. 26003812 PMC4856150

[22] Eid SM, Abougergi MS, Albaeni A, Chandra-Strobos N. Survival, expenditure and disposition in patients following out-of-hospital cardiac arrest: 1995–2013. Resuscitation. 2017;113:13–20. Epub 2017/01/21. doi: 10.1016/j.resuscitation.2016.12.027 . 28104426

[23] Coppler PJ, Elmer J, Rittenberger JC, Callaway CW, Wallace DJ. Demographic, social, economic and geographic factors associated with long-term outcomes in a cohort of cardiac arrest survivors. Resuscitation. 2018;128:31–6. Epub 2018/05/01. doi: 10.1016/j.resuscitation.2018.04.032 ; PubMed Central PMCID: PMC6004263. 29705340 PMC6004263

[24] Cho KH, Nam CM, Lee EJ, Choi Y, Yoo KB, Lee SH, et al. Effects of individual and neighborhood socioeconomic status on the risk of all-cause mortality in chronic obstructive pulmonary disease: A nationwide population-based cohort study, 2002–2013. Respir Med. 2016;114:9–17. Epub 2016/04/26. doi: 10.1016/j.rmed.2016.03.003 . 27109806

[25] Choi Y, Kwon IH, Jeong J, Chung J, Roh Y. Incidence of Adult In-Hospital Cardiac Arrest Using National Representative Patient Sample in Korea. Healthc Inform Res. 2016;22(4):277–84. Epub 2016/11/30. doi: 10.4258/hir.2016.22.4.277 ; PubMed Central PMCID: PMC5116539. 27895959 PMC5116539

[26] Kim TH, Ro YS, Shin SD, Song KJ, Hong KJ, Park JH, et al. Association of health insurance with post-resuscitation care and neurological outcomes after return of spontaneous circulation in out-of-hospital cardiac arrest patients in Korea. Resuscitation. 2019;135:176–82. Epub 2019/01/15. doi: 10.1016/j.resuscitation.2018.12.023 . 30639790

[27] Kim C, Choi HJ, Moon H, Kim G, Lee C, Cho JS, et al. Prehospital advanced cardiac life support by EMT with a smartphone-based direct medical control for nursing home cardiac arrest. The American journal of emergency medicine. 2019;37(4):585–9. Epub 2018/07/14. doi: 10.1016/j.ajem.2018.06.031 . 30001817

[28] Park TK, Gwag HB, Park SJ, Park H, Kang D, Park J, et al. Differential prognosis of vasospastic angina according to presentation with sudden cardiac arrest or not: Analysis of the Korean Health Insurance Review and Assessment Service. International journal of cardiology. 2018;273:39–43. Epub 2018/10/05. doi: 10.1016/j.ijcard.2018.09.082 . 30282600

[29] Sundararajan V, Quan H, Halfon P, Fushimi K, Luthi JC, Burnand B, et al. Cross-national comparative performance of three versions of the ICD-10 Charlson index. Medical care. 2007;45(12):1210–5. Epub 2007/11/17. doi: 10.1097/MLR.0b013e3181484347 . 18007172

[30] Global, regional, and national life expectancy, all-cause mortality, and cause-specific mortality for 249 causes of death, 1980–2015: a systematic analysis for the Global Burden of Disease Study 2015. Lancet (London, England). 2016;388(10053):1459–544. Epub 2016/10/14. doi: 10.1016/s0140-6736(16)31012-1 ; PubMed Central PMCID: PMC5388903. 27733281 PMC5388903

[31] Quan H, Khan N, Hemmelgarn BR, Tu K, Chen G, Campbell N, et al. Validation of a case definition to define hypertension using administrative data. Hypertension. 2009;54(6):1423–8. Epub 2009/10/28. doi: 10.1161/HYPERTENSIONAHA.109.139279 . 19858407

[32] Khokhar B, Jette N, Metcalfe A, Cunningham CT, Quan H, Kaplan GG, et al. Systematic review of validated case definitions for diabetes in ICD-9-coded and ICD-10-coded data in adult populations. BMJ open. 2016;6(8):e009952. Epub 2016/08/09. doi: 10.1136/bmjopen-2015-009952 ; PubMed Central PMCID: PMC4985868. 27496226 PMC4985868

[33] Pace R, Peters T, Rahme E, Dasgupta K. Validity of Health Administrative Database Definitions for Hypertension: A Systematic Review. Can J Cardiol. 2017;33(8):1052–9. Epub 2017/07/30. doi: 10.1016/j.cjca.2017.05.025 . 28754391

[34] National Health Insurance System of Korea. National Health Insurance Service, 2007.

[35] Austin PC. Balance diagnostics for comparing the distribution of baseline covariates between treatment groups in propensity-score matched samples. Stat Med. 2009;28(25):3083–107. Epub 2009/09/17. doi: 10.1002/sim.3697 ; PubMed Central PMCID: PMC3472075. 19757444 PMC3472075

[36] Population for Korea. Statistics Korea. Korea National Census database. Korean Statistical Information Service. 2010. http://kosis.kr/statHtml/statHtml.do?orgId=101&tblId=DT_1BPA002&conn_path=I2.

[37] Fowler-Brown A, Corbie-Smith G, Garrett J, Lurie N. Risk of cardiovascular events and death—does insurance matter? J Gen Intern Med. 2007;22(4):502–7. Epub 2007/03/21. doi: 10.1007/s11606-007-0127-2 ; PubMed Central PMCID: PMC1829431. 17372800 PMC1829431

[38] Pancholy S, Patel G, Pancholy M, Nanavaty S, Coppola J, Kwan T, et al. Association Between Health Insurance Status and In-Hospital Outcomes After ST-Segment Elevation Myocardial Infarction. The American journal of cardiology. 2017;120(7):1049–54. Epub 2017/08/22. doi: 10.1016/j.amjcard.2017.06.041 . 28823480

[39] Reinier K, Thomas E, Andrusiek DL, Aufderheide TP, Brooks SC, Callaway CW, et al. Socioeconomic status and incidence of sudden cardiac arrest. CMAJ: Canadian Medical Association journal = journal de l’Association medicale canadienne. 2011;183(15):1705–12. Epub 2011/09/14. doi: 10.1503/cmaj.101512 ; PubMed Central PMCID: PMC3193117. 21911550 PMC3193117

[40] Kurz MC, Donnelly JP, Wang HE. Variations in survival after cardiac arrest among academic medical center-affiliated hospitals. PloS one. 2017;12(6):e0178793. Epub 2017/06/06. doi: 10.1371/journal.pone.0178793 ; PubMed Central PMCID: PMC5459445. 28582400 PMC5459445

[41] Høybye M, Stankovic N, Holmberg M, Christensen HC, Granfeldt A, Andersen LW. In-Hospital vs. Out-of-Hospital Cardiac Arrest: Patient Characteristics and Survival. Resuscitation. 2021;158:157–65. doi: 10.1016/j.resuscitation.2020.11.016 33221361

[42] Kagawa E, Inoue I, Kawagoe T, Ishihara M, Shimatani Y, Kurisu S, et al. Assessment of outcomes and differences between in- and out-of-hospital cardiac arrest patients treated with cardiopulmonary resuscitation using extracorporeal life support. Resuscitation. 2010;81(8):968–73. doi: 10.1016/j.resuscitation.2010.03.037 20627526

[43] Fredriksson M, Aune S, Bång A, Thorén AB, Lindqvist J, Karlsson T, et al. Cardiac arrest outside and inside hospital in a community: mechanisms behind the differences in outcome and outcome in relation to time of arrest. Am Heart J. 2010;159(5):749–56. doi: 10.1016/j.ahj.2010.01.015 20435182

[44] Dumas F, Bougouin W, Geri G, Lamhaut L, Bougle A, Daviaud F, et al. Is epinephrine during cardiac arrest associated with worse outcomes in resuscitated patients? J Am Coll Cardiol. 2014;64(22):2360–7. doi: 10.1016/j.jacc.2014.09.036 25465423

[45] Carr BG, Kahn JM, Merchant RM, Kramer AA, Neumar RW. Inter-hospital variability in post-cardiac arrest mortality. Resuscitation. 2009;80(1):30–4. doi: 10.1016/j.resuscitation.2008.09.001 18952359

[46] Fukuda T, Yasunaga H, Horiguchi H, Ohe K, Fushimi K, Matsubara T, et al. Health care costs related to out-of-hospital cardiopulmonary arrest in Japan. Resuscitation. 2013;84(7):964–9. Epub 2013/03/09. doi: 10.1016/j.resuscitation.2013.02.019 . 23470473

