# Supplementary material for: Impact of insurance type on outcomes in cardiac arrest patients from 2004 to 2015: A nation-wide population-based study
Source: PLoS One. 2021 Jul 14;16(7):e0254622. doi: 10.1371/journal.pone.0254622 (PMC8279316; doi:10.1371/journal.pone.0254622)
Supplement: S1 Table — (DOCX) [file pone.0254622.s001.docx]

**S1 Table. Characteristics, hospital factors, treatments, and outcomes according to type of insurance coverage in cardiac arrest patients admitted through emergency room**

|  | **Total**  **N (%)** | **NHI**  **N (%)** | **MA**  **N (%)** | *p*-value |
| --- | --- | --- | --- | --- |
| **Age**, years  Mean ± SD**^†^** | 65.9 ± 15.5 | 65.6 ± 15.5 | 65.7 ± 15.7 | <.0001^a^ |
| **Gender,** Male, n (%) | 208445 (62.0) | 186995 (62.9) | 21450 (55.3) | <.0001^b^ |
| **Urbanization level**, n (%) | |  |  |  |
| Urban | 286158 (85.3) | 254380 (85.8) | 31778 (82.0) | <.0001^b^ |
| Rural | 49195 (14.7) | 42233 (14.2) | 6962 (18.0) |  |
| **Level of Hospital**, n (%) | |  |  |  |
| Tertiary | 119578 (35.6) | 109248 (36.8) | 10330 (26.6) | <.0001^c^ |
| Secondary | 186933 (55.6) | 163105 (54.9) | 23828 (61.4) |  |
| Primary | 29535 (8.8) | 24904 (8.4) | 4631 (11.9) |  |
| **Capacity of hospital**, Beds, n (%) | |  |  |  |
| <300 | 86453 (25.7) | 73722 (24.8) | 12731 (32.8) | <.0001^c^ |
| 300-499 | 66066 (19.7) | 57923 (19.5) | 8143 (21.0) |  |
| 500-799 | 117446 (35.0) | 104988 (35.3) | 12458 (32.1) |  |
| >800 | 66081 (19.7) | 60624 (20.4) | 5457 (14.1) |  |
| **CCI**^*^ < 2 | 145437 (43.3) | 133922 (45.1) | 11515 (29.7) | <.0001^b^ |
| **Comorbidities,** n (%) | |  |  |  |
| Cancer | 54470 (16.2) | 48801 (16.4) | 5669 (14.6) | <.0001^b^ |
| Ischemic Stroke | 53662 (16.0) | 45490 (15.3) | 8172 (21.1) | <.0001^b^ |
| Hemorrhagic Stroke | 8187 (2.4) | 6848 (2.3) | 1339 (3.45) | <.0001^b^ |
| Myocardial infarction | 11880 (3.5) | 10365 (3.5) | 1515 (3.9) | <.0001^b^ |
| Angina | 45602 (13.6) | 40065 (13.5) | 5537 (14.3) | <.0001^b^ |
| Heart failure | 32882 (9.8) | 28247 (9.5) | 4635 (12.0) | <.0001^b^ |
| Arrhythmia | 28516 (8.5) | 24973 (8.4) | 3543 (9.1) | <.0001^b^ |
| Hypertension | 128625 (38.3) | 113915 (38.3) | 14710 (37.9) | 0.1284^b^ |
| Diabetes Mellitus | 58183 (17.3) | 50745 (17.1) | 7438 (19.2) | <.0001^b^ |
| Lipidemia | 77980 (23.2) | 67856 (22.8) | 10124 (26.1) | <.0001^b^ |
| Chronic Pulmonary disease | 91076 (27.1) | 77836 (26.2) | 13240 (34.1) | <.0001^b^ |
| Chronic Renal Failure | 22791 (6.8) | 18652 (6.3) | 4139 (10.7) | <.0001^b^ |
| Hemodialysis | 11587 (3.5) | 9376 (3.2) | 2211 (5.7) | <.0001^b^ |
| Liver cirrhosis | 57691 (17.2) | 48083 (16.2) | 9608 (24.8) | <.0001^b^ |
| Defibrillation | 98386 (29.3) | 88229 (29.7) | 10157 (26.2) | <.0001^b^ |
| Epinephrine | 287708 (85.6) | 254888 (85.8) | 32820 (84.6) | <.0001^b^ |
| Amiodarone | 44848 (13.4) | 40649 (13.7) | 4199 (10.8) | <.0001^b^ |
| Atropine | 208808 (62.1) | 184677 (62.1) | 24131 (62.2) | 0.7487^b^ |
| CAG^†^ | 21190 (6.3) | 19558 (6.6) | 1632 (4.2) | <.0001^b^ |
| PCI^‡^ | 13051(3.9) | 12083 (4.1) | 968 (2.5) | <.0001^b^ |
| CABG**^§^** | 707 (0.2) | 658 (0.2) | 49 (0.1) | <.0001^b^ |
| VA-ECMO**^\|\|^** | 3144 (0.9) | 2998 (1.0) | 146 (0.4) | <.0001^b^ |
| ICD**^#^** | 902 (0.3) | 864 (0.3) | 38 (0.1) | <.0001^b^ |
| CRRT**^**^** | 19418 (5.8) | 17288 (5.8) | 2130 (5.5) | 0.010^b^ |
| Brain CT | 82855 (24.7) | 73349 (24.7) | 9506 (24.5) | 0.4694^b^ |
| Brain MRI | 14029 (4.2) | 12491 (4.2) | 1538 (4.0) | 0.0281^b^ |
| EEG^¶^ | 16169 (4.8) | 14518 (4.9) | 1651 (4.3) | <.0001^b^ |
| Therapeutic hypothermia | 4485 (1.3) | 4205 (1.4) | 280 (0.7) | <.0001^b^ |
| 30-ds death | 287160 (85.5) | 253920 (85.4) | 33240 (85.7) | <.0001^b^ |
| 6-mo death | 303307 (90.3) | 267532 (90.0) | 35775 (92.2) | <.0001^b^ |
| 1-yr death | 333442 (99.2) | 269889 (90.8) | 36185 (93.3) | <.0001^b^ |
| Total | 336046 (100) | 297257 | 38789 |  |

^a^Student`s t-test; ^b^Fisher`s exact test; ^c^Chi-square test

CCI^*^, Charlson Comorbidity Index; CAG^†^, Coronary angiography; PCI^‡^, Percutaneous coronary intervention; CABG**^§^**, Coronary artery bypass graft; VA-ECMO**^||^**, Veno-arterial extracorporeal membrane oxygenation; ICD**^#^**, Implanted cardioverter-defibrillator; CRRT**^**^**, Continuous renal replacement therapy; EEG^¶^, Electroencephalography
