# Supplementary material for: Impact of insurance type on outcomes in cardiac arrest patients from 2004 to 2015: A nation-wide population-based study
Source: PLoS One. 2021 Jul 14;16(7):e0254622. doi: 10.1371/journal.pone.0254622 (PMC8279316; doi:10.1371/journal.pone.0254622)
Supplement: S2 Table — (DOCX) [file pone.0254622.s002.docx]

**S2 Table. The characteristics, comorbidities, and mortality between both groups by admission route**

| n (%) | Admission  Through Not ER^*^ | Admission  Through ER |
| --- | --- | --- |
| Age, years | 69.9±14.4 | 65.9±15.5 |
| Gender, Male | 85236 (56.2) | 208735 (62.0) |
| Insurance type |  |  |
| National Health Insurance | 125210 (82.6) | 297257 (88.5) |
| Medical aid | 26186 (17.3) | 38789 (11.5) |
| Urbanization level |  |  |
| Urban | 124432 (82.2) | 286436 (85.3) |
| Rural | 26849 (17.8) | 49204 (14.7) |
| Charlson comorbidity index < 2 | 33425 (22.1) | 145716 (43.3) |
| Cancer | 46199 (30.5) | 54522 (16.2) |
| Ischemic Stroke | 38461 (25.4) | 53720 (15.9) |
| Hemorrhagic Stroke | 9662 (6.4) | 8197 (2.4) |
| Myocardial infarction | 7354 (4.9) | 11899 (3.5) |
| Angina | 22711 (14.9) | 45665 (13.6) |
| Heart failure | 19254 (12.7) | 32923 (9.8) |
| Arrhythmia | 16883 (11.1) | 28547 (8.5) |
| Hypertension | 88876 (58.6) | 169667 (50.4) |
| Hypertension+ medication | 54333 (35.8) | 128754 (38.3) |
| Diabetes Mellitus | 54434 (35.9) | 100996 (30.0) |
| Diabetes Mellitus + medication | 24017 (15.8) | 58235 (17.3) |
| Lipidemia | 35959 (23.7) | 78058 (23.2) |
| Chronic pulmonary disease | 55025 (36.3) | 91176 (27.1) |
| Chronic Renal Failure | 14907 (9.8) | 22813 (6.8) |
| Hemodialysis | 9368 (6.2) | 11602 (3.5) |
| Live cirrhosis | 32356 (21.3) | 57758 (17.2) |
| Defibrillation | 30729 (20.3) | 98559 (29.3) |
| Epinephrine | 108575 (71.6) | 288056 (85.6) |
| 30-ds death | 124299 (81.9) | 287298 (85.4) |
| 6-mo death | 139012 (91.7) | 303486 (90.2) |
| 1-yr death | 140440 (92.6) | 306229 (91.0) |
| Total | 151606 | 336523 |

^*^ED, Emergency room
